# Comparison Combination of Autologous Fibroblast Cells Plus Platelet Rich Plasma (PRP) With PRP Alone in Treatment of Atrophic Acne Scars, a Split‐Face Pilot Study With Biometric Assessment

**DOI:** 10.1111/jocd.70413

**Published:** 2025-08-25

**Authors:** Sona Zare, Roya Zeinali, Maryam Nouri, Abbas Dehghani, Solmaz Zare, Sharin Khatib, Mohammad Ali Nilforoushzadeh

**Affiliations:** ^1^ Skin and Stem Cell Research Center Tehran University of Medical Sciences Tehran Iran; ^2^ Pars Fundamental Bio Structure Company Sharif University of Technology Tehran Iran; ^3^ Stem Cell and Regenerative Medicine Institute, Sharif University of Technology Tehran Iran; ^4^ Department of Mechanical Engineering Sharif University of Technology Tehran Iran; ^5^ Department of Dermatology, School of Medicine, Hazrat Fatemeh Hospital Iran University of Medical Sciences Tehran Iran; ^6^ Laser Application in Medical Sciences Research Center Shahid Beheshti University of Medical Sciences Tehran Iran; ^7^ Faculty of Veterinary Medicine Islamic Azad University Science and Research Branch Tehran Iran; ^8^ Skin Repair Research Center, Jordan Dermatology and Hair Transplantation Center Tehran Iran

**Keywords:** cell therapy, fibroblast, platelet rich plasmaatrophic acne scars

## Abstract

**Introduction:**

Acne scars are a common side effect of acne vulgaris that can negatively impact patients' quality of life and contribute to depression and anxiety. There is no definitive treatment for this condition. Ongoing research aims to discover better therapeutic options for acne scars. This study was conducted to assess the efficacy and safety of autologous fibroblast injections with and without platelet‐rich plasma (PRP) for this condition.

**Method:**

In this split‐face pilot study, eight patients with atrophic acne scars were injected with a PRP‐fibroblast suspension on one side of their face, while the other side received only PRP as a control, in three biweekly sessions. Patients were assessed before treatment and 6 months later based on biometric indices, including skin ultrasound, hydration level, skin elasticity (R2, R5, and R7), the number of pores and spots, and the acne scar's volume, area, and depth.

**Results:**

The median differences in skin thickness before and after follow‐up were 32.57 on the treatment side and 5.33 on the control side. These changes were significantly greater on the treatment side (*p*‐value: 0.018). Improvements in skin elasticity (*p*‐value: 0.012), skin hydration level (*p*‐value: 0.028), the number of fine pores (*p*‐value: 0.043), and the volume and area of scars (*p*‐value: 0.043) were significantly better with the combination therapy. While the number of large pores and spots also improved significantly, the extent of improvement was comparable to that observed with PRP alone.

**Conclusion:**

The PRP‐fibroblast suspension may be considered a novel and effective therapeutic approach for atrophic acne scars.

## 1 Introduction

Acne vulgaris is a prevalent inflammatory skin disorder most commonly observed in adolescence, affecting nearly 90% of individuals with varying degrees of severity [1]. Even after resolution, this condition can result in skin scars and deformities, negatively impacting patients' quality of life and contributing to depression and anxiety [2, 3].

Atrophic acne scars, which result primarily from skin collagen loss, are traditionally classified into three types based on morphology: icepick, boxcar, and rolling scars. Less commonly, hypertrophic scars and keloids may develop, particularly in individuals with darker skin tones [4]. Treatment strategies for acne scars vary and are often tailored to the type and severity of the scars, as well as the patient's skin phenotype. There is no single definitive treatment protocol for this condition. Ongoing research aims to identify the most effective modalities. Currently, microneedling and laser treatments are commonly considered first‐line therapies [5, 6].

Platelet‐rich plasma (PRP) is an established method that has demonstrated efficacy in treating acne scars in several studies [7, 8, 9, 10, 11]. PRP has also been shown to have positive effects on dermal fibroblast proliferation [12].

Skin augmentation techniques for acne scars have been widely used over the past two decades, beginning with bovine collagen, which was the most popular injectable implant in the United States for nearly 25 years [13]. Since then, human bioengineered collagen products and hyaluronic acid‐containing fillers have become available and commonly used in the treatment of acne scars. Although these fillers provide immediate esthetic results, their effects are not long‐lasting due to gradual reabsorption, and their long‐term efficacy remains uncertain in the literature [14]. Furthermore, they may be associated with adverse reactions, including erythematous pruritic lesions, granulomatous inflammation, and hypersensitivity responses [15, 16]. In contrast, autologous fibroblast injections represent a regenerative and potentially safer alternative. They are less likely to cause these complications and may offer more durable improvements in acne scarring.

Alternatively, fibroblast cells, which are crucial components of connective tissue and responsible for synthesizing collagen and other extracellular matrix components, have been utilized for contour deformity correction and skin rejuvenation [17, 18, 19]. It is hypothesized that the stimulation of recipient fibroblast cells for better collagen synthesis is the main treatment mechanism [20].

Given the scarcity of clinical trials on this subject, this study was conducted to assess the efficacy and safety of a combination therapy involving autologous fibroblast transplantation and PRP injection for atrophic acne scar treatment and to compare it with PRP treatment alone.

## 2 Methods

### 2.1 Patient Selection

This pilot study was conducted on eight patients with acne scars from March 2018 to Feb 2019 at Skin and Stem Cell Research Center of Tehran University of Medical Sciences. The inclusion criteria for the study were: (1) age between 20 and 59, (2) exhibiting atrophic acne scars on their faces without active acne lesions, and (3) willingness to complete the study. Exclusion criteria included: (1) history of coagulation disorders, (2) pregnancy or breastfeeding, (3) signs or symptoms of infection (local or systemic), (4) concurrent skin disorders, (5) history of systemic diseases, (6) positive HIV or hepatitis tests, (7) mental health disorders, and (8) tobacco or alcohol use.

Prior to participation, all individuals provided written informed consent, confirming their understanding of the therapeutic procedure.

### 2.2 Randomization and Blinding

All eight patients had atrophic acne scars on their faces, which were divided into two sections. One side was randomly assigned to receive platelet‐rich plasma (PRP) alone (control side), while the other was treated with a combination of PRP and fibroblasts (case side). The allocation of each patient's facial sides was determined by a computer‐generated randomization list. In this study, both the patients (using identical syringes) and the statistical expert were blinded to the specific treatment administered to each side.

### 2.3 Isolation and Preparation of Fibroblasts

A 5‐mm punch biopsy was obtained from the retro‐auricular area to collect the skin sample. The sample was then transferred to a tube containing Dulbecco's Modified Eagle Medium (DMEM) with high glucose (ATCDH‐883; Atocel, Austria) and kept at 4°C. The specimen was sterilized by washing it in 70% alcohol and a solution of phosphate‐buffered saline (PBS) (ATDP1‐850; Atocel, Austria).

**FIGURE 1:**
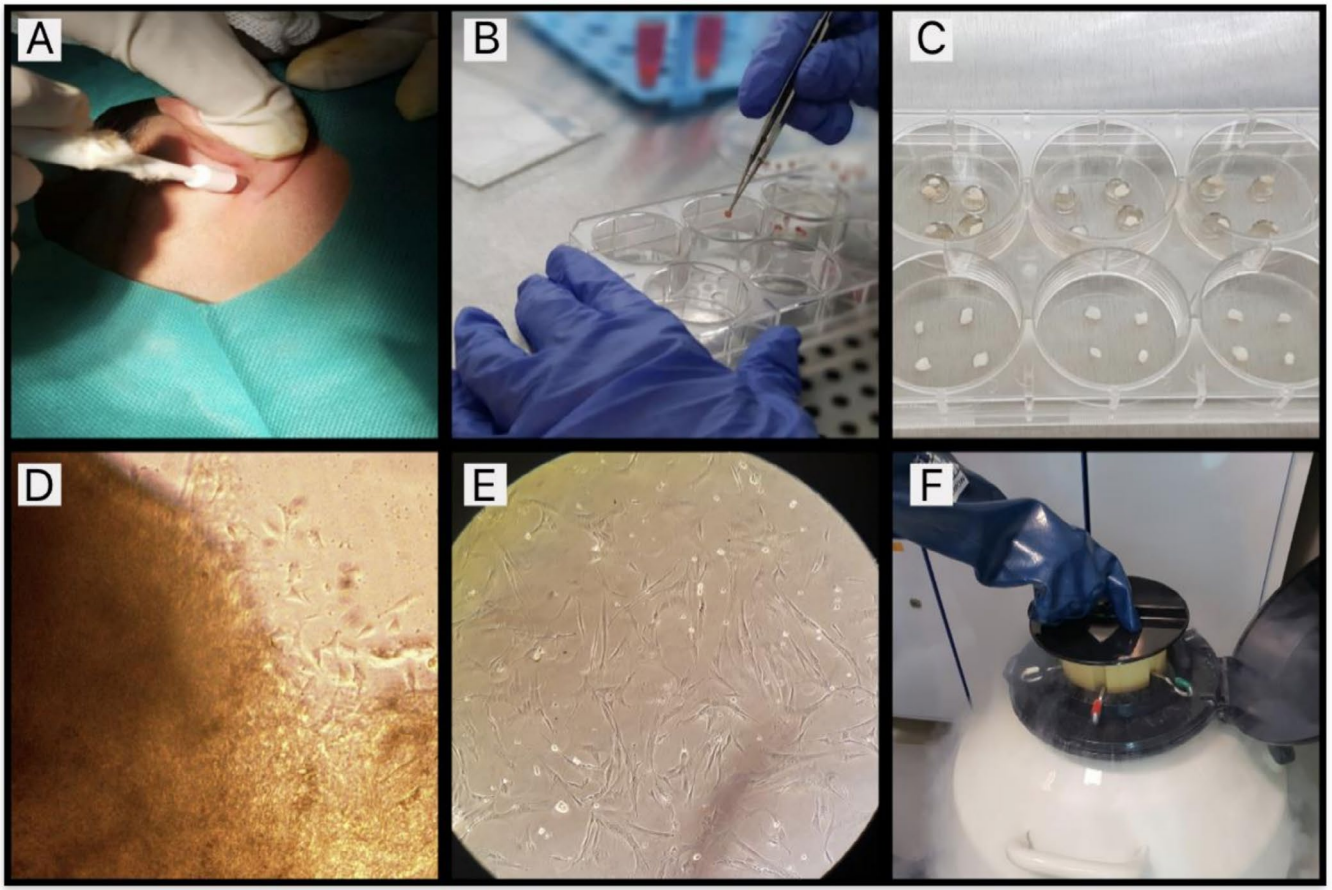
Fibroblast isolation method used in the current study. Punch biopsy from the retro‐auricular area (A); transfer of the sample to Dulbecco's Modified Eagle Medium (DMEM) (B); explantation technique (C); fibroblast cells 6 days after isolation (D); fibroblast cells after the third passage (E); cryopreservation of cells at passage four using liquid nitrogen (F).

Following this, fibroblasts were cultured using the explantation technique in a different culture medium, which consisted of DMEM and 10% fetal human serum (ATSM‐15; Atocel, Austria). Fibroblasts were passaged with 0.25% trypsin when they reached 80% confluency. These cells were subsequently frozen at passage four using liquid nitrogen. Quality control tests were performed before freezing the fibroblasts and with each injection at passage three (Figure 1). Prior to injection, the fibroblasts were suspended in human PRP in the 3 cc syringe.

### 2.4 PRP Preparation

**FIGURE 2:**
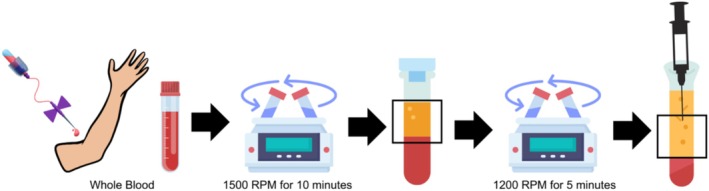
Illustration of the PRP preparation method used in the current study.

The preparation of PRP followed a two‐step centrifugation process (Figure 2). Initially, after sterilizing the antecubital fossa, 20 mL of whole blood were drawn from each participant and divided equally into two sterile tubes (Pars fundamental bio structure company, Tehran, Iran), each containing 1.5 mL of ACDA (acid citrate dextrose anticoagulant). The first centrifugation was performed at 1500 rpm for 10 min using a Hettich Universal 320 centrifuge at room temperature, separating red and white blood cells from plasma and platelets. In the second centrifugation step, the plasma and platelets, excluding red blood cells, were transferred to new tubes and centrifuged again for 5 min at 1200 rpm. This process concentrated the platelets, producing PRP that was approximately 3–4 times more concentrated than whole blood. The PRP was then drawn from the tubes and placed into 1 mL insulin syringes, with each participant receiving 3 cc of activated PRP.

### 2.5 Intervention Methods

The patients were anesthetized, receiving topical lidocaine/prilocaine 2.5% (Xyla‐P, Tehran Chemie Pharmaceuticals, Iran) about 20 min. Thereafter, the acne scar area was cleansed using 70% alcohol swabs. 3 cc PRP‐fibroblasts suspension was injected intradermally at depression sites of atrophic scar inside the prepared zone using a 30G needle till the lentil formation and blanching. The same procedure was done for the control side of the face, using only 3 cc PRP solution. This process was performed in three sessions with an interval of 2 weeks.

### 2.6 Assessment Methods

All patients were evaluated at baseline and 6 months later based on the objective evaluation with biometric indices using VisioFace (Courage + Khazaka, Cologne, Germany) including (a) the hydration level of the skin surface (stratum corneum) by Multi Probe Adapter 9 (MPA‐9) Corneometer, (b) the trans‐epidermal water loss (TEWL) by MPA‐9 Tewameter, (c) melanin index by Mexameter, (d) erythema by Mexameter, (e) the skin elasticity (R2 [the ability to return to the original position], R5 [the elastic part of the suction phase], and R7 [the portion of the elastic recovery compared to the complete curve]) by Cutometer [21], (f) the skin thickness by ultrasonography [22], (g) the skin density by ultrasonography, (h) number of pores and spots by VisioFace, and (i) acne scar's volume, area, and depth by VisioFace.

### 2.7 Sample Size

The sample size was calculated using the following formulae: $n=2\left({\left(Z_{\alpha }+Z_{\beta }\right)}^{2}\right)\sigma^{2}*\left(1-r\right)/d^{2}$, in which *α* was set to 0.05, *β* to 0.2, and *r* to 0.5. Finally, we calculated eight patients are needed to be included in the trial.

### 2.8 Statistical Analysis

All data were analyzed using a statistical package for social sciences version 25 (SPSS 25). Due to the small sample size and assumed non‐normal data distribution, nonparametric methods were employed. Median, first quartile (Q1), and third quartile (Q3) were reported for all variables. The Wilcoxon signed‐rank test was used to assess changes within groups, and the Mann–Whitney *U* test was used for between‐group comparisons. A *p*‐value of < 0.05 was considered statistically significant. Additionally, a post hoc power analysis was conducted, confirming that the study achieved sufficient statistical power (> 80%) for key parameters.

## 3 Results

This pilot study enrolled eight patients (six females, two males) with a mean age of 41.88 ± 9.57 years (ranging from 34 to 59).

In the control group, statistically significant changes were observed in trans‐epidermal water loss (TWL) (*p* = 0.028), color (*p* = 0.042), R5 (*p* = 0.017), all sonographic parameters except for epidermal and dermal thickness, pores (*p* = 0.043), and scar acne volume and area (both *p* = 0.043) (Table S1).

In the case group, significant mean changes were also noted in TWL (*p* = 0.028), color (*p* = 0.027), R5 (*p* = 0.017), all sonographic parameters except for epidermal thickness, pores (*p* = 0.043), and scar acne volume and area (both *p* = 0.043) (Table S2).

**FIGURE 3:**
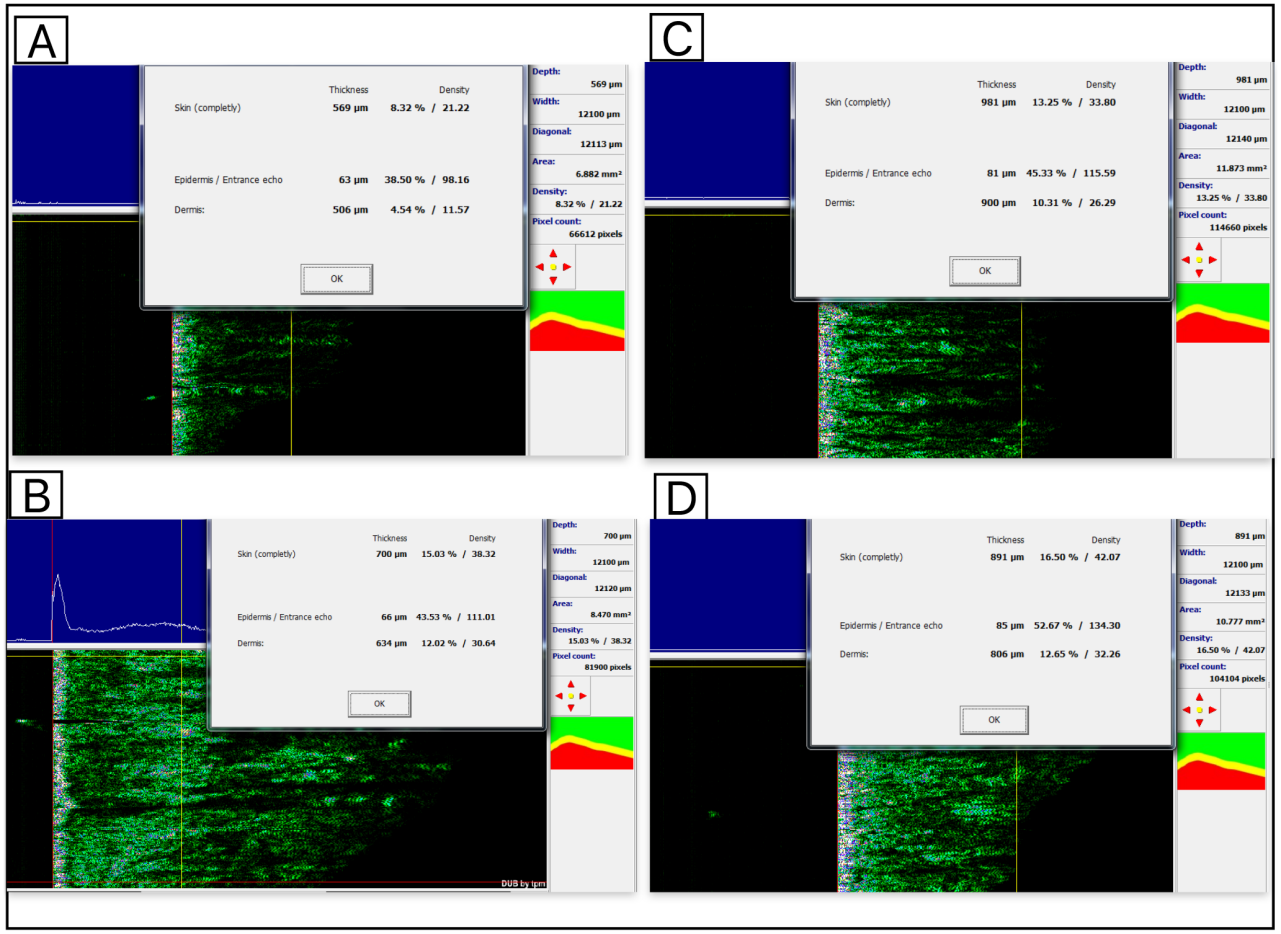
The sonographic study of a patient before and after treatment; before treatment with fibroblasts and PRP injection (A), before treatment with PRP injection (B), after treatment with fibroblasts and PRP injection (C), after treatment with PRP injection (D).

Posttreatment comparisons between the groups showed significant improvements in sonographic indices on the case side compared to the control (*p* < 0.05) (Figure 3, Table 1).

**TABLE 1:** The changes in and between groups regarding the change's percentage.

	Group	Percentiles			*p*
		25	50 (Median)	75	
TEWL	Case	−43.50	−27.25	−20.10	0.018*
	Control	−35.66	−21.16	−13.79	
HLSS	Case	−43.98	9.00	22.66	0.028*
	Control	−49.39	3.42	14.36	
Melanin	Case	−18.34	−4.39	13.09	0.018*
	Control	−14.40	−2.58	13.87	
Erythema	Case	−14.61	−4.78	21.68	0.018*
	Control	−10.60	−2.43	22.30	
Color	Case	0.00	15.38	52.17	0.345
	Control	0.00	7.69	34.78	
R2	Case	−8.95	3.69	21.22	0.012*
	Control	−10.54	2.35	15.44	
R5	Case	13.38	25.21	48.79	0.012*
	Control	11.44	17.94	44.07	
R7	Case	0.73	10.89	31.62	0.012*
	Control	0.07	8.49	18.05	
Skin thickness	Case	4.13	32.57	65.41	0.018*
	Control	0.09	5.33	24.20	
Epidermal thickness	Case	0.00	13.64	28.57	0.018*
	Control	−1.22	3.03	15.38	
Dermal thickness	Case	7.29	27.60	70.29	0.028*
	Control	−0.10	5.60	26.74	
Skin density	Case	12.24	19.51	59.25	0.018*
	Control	0.31	6.93	23.20	
Epidermal density	Case	17.74	23.96	29.58	0.018*
	Control	6.42	8.07	12.39	
Dermal density	Case	9.81	23.96	104.36	0.018*
	Control	0.99	7.74	27.06	
Fine pore	Case	−56.91	−52.49	−30.78	0.043*
	Control	−29.52	−16.67	−7.26	
Large pore	Case	−69.11	−60.00	−30.00	0.345
	Control	−63.17	−52.08	−25.24	
Spot	Case	−90.00	−66.67	−15.00	1.000
	Control	−83.33	−50.00	−25.00	
Scar acne volume	Case	−51.96	−43.33	−25.66	0.043*
	Control	−36.81	−28.33	−16.82	
Scar acne area	Case	−51.90	−46.66	−26.42	0.043*
	Control	−31.32	−25.53	−22.57	
Scar acne depth	Case	0.00	0.00	5.56	0.713
	Control	−10.00	−10.00	12.50	

Abbreviations: HLSS, hydration level of skin surface; R2, the ability to return to the original position; R5, the elastic part of the suction phase; R7, the portion of the elastic recovery compared to the complete curve; TEWL, trans‐epidermal water loss.

* A *p*‐value of < 0.05 was considered statistically significant.

Superior outcomes in R2, R5, and R7 were observed on the case side (*p* = 0.012 for all). Although trans‐epidermal water loss decreased across the entire face, the reduction was significantly more pronounced in the area treated with fibroblast + PRP (*p* = 0.018).

MPA‐9 showed that the hydration level of the stratum corneum increased on both sides; but this enhancement was greater on the case side (*p*‐value: 0.028).

The reduction in the melanin and erythema index was higher on the case side; which was statistically significant (*p*‐value: 0.018 for both).

**FIGURE 4:**
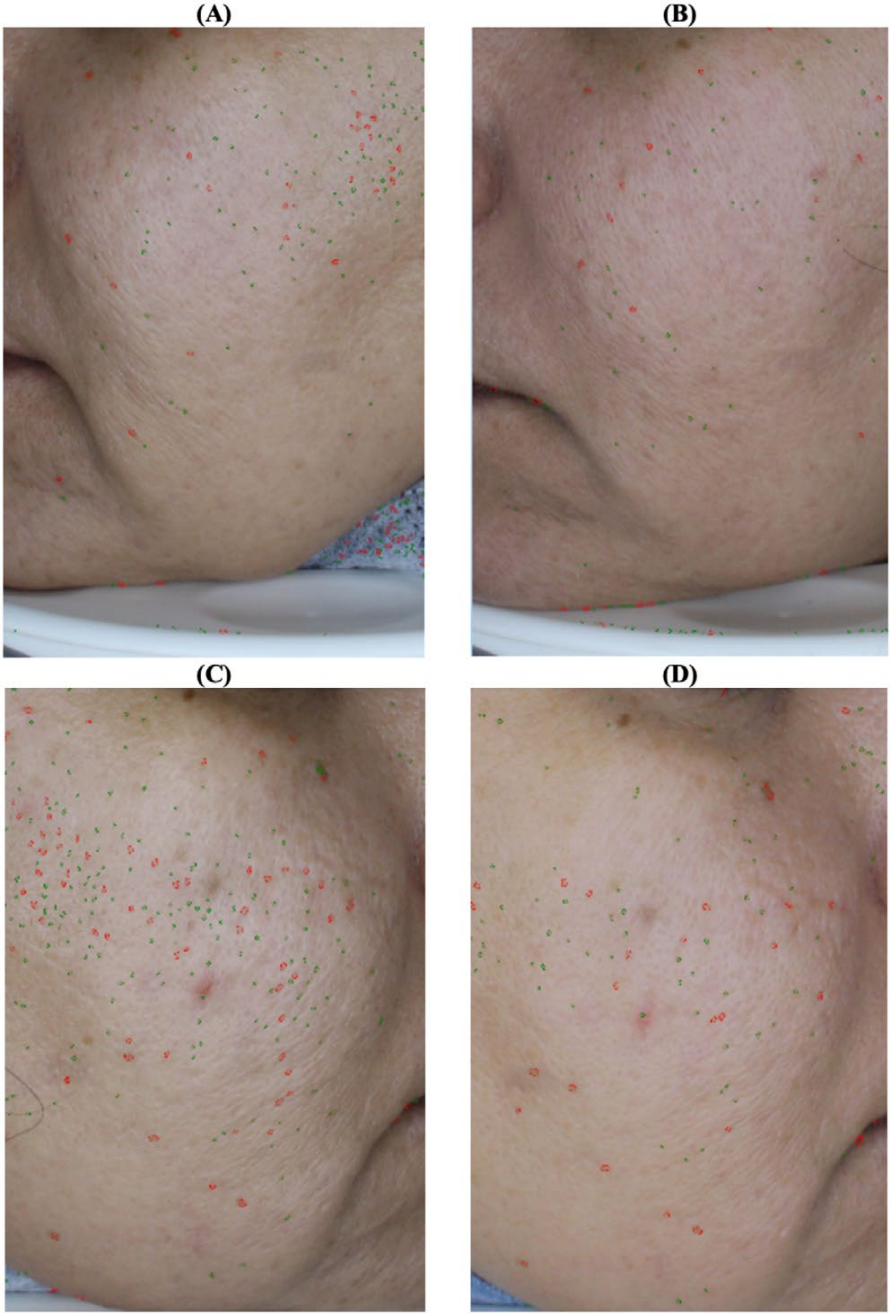
Visioface study of the pores; (A) the left side of the face before the treatment with PRP; (B) the left side of the face after the treatment with PRP; (C) the right side of the face before the treatment with PRP and fibroblasts; and (D) the right side of the face after the treatment with PRP and fibroblasts.

**FIGURE 5:**
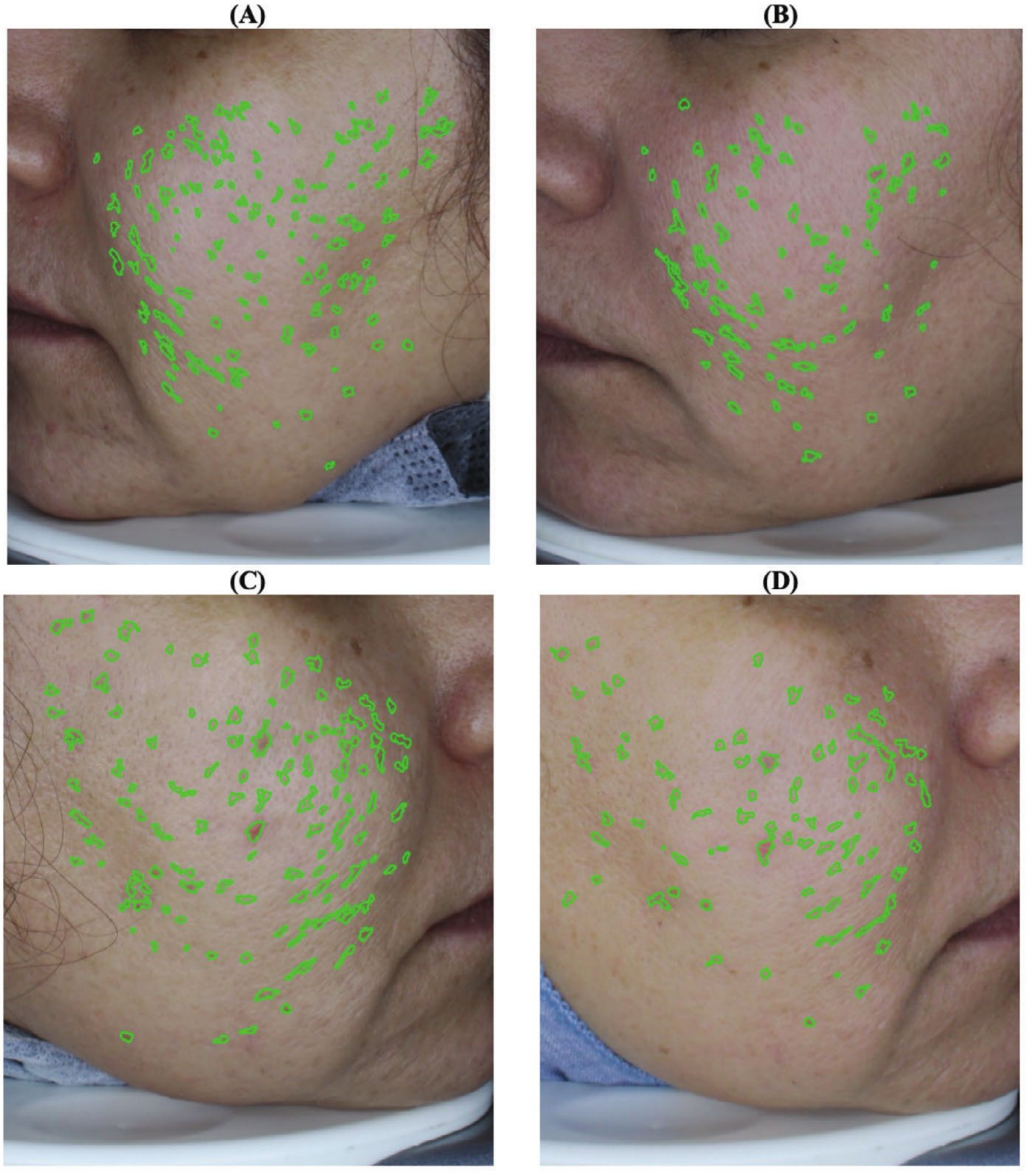
Visioface study of the scar acne volume; (A) the left side of the face before the treatment with PRP, (B) the left side of the face after the treatment with PRP, (C) the right side of the face before the treatment with PRP and fibroblasts, and (D) the right side of the face after the treatment with PRP and fibroblasts.

A more notable decline in the number of fine pores was observed on the case side (*p* = 0.043) (Figure 4); while reductions in large pores were similar on both sides. Scar acne volume and area improved bilaterally, with superior outcomes on the side receiving combination therapy (*p* = 0.043) (Figure 5).

According to the post hoc power analysis for changes between groups, only the fine pore changes demonstrated a strong chance of detecting a real effect (power > 80%); Scar Acne Area showed moderate power (50%–80%). Other parameters exhibited low power (< 50%), indicating a higher risk of Type II error and suggesting that the study was underpowered for those outcomes.

## 4 Discussion

According to biometric assessments of this experimental study, three bi‐weekly injections of both fibroblast‐PRP suspension and PRP alone showed improvement in atrophic acne scars; however, the combination of fibroblasts and PRP significantly enhanced acne scars more than PRP alone.

The use of PRP for acne scar improvement is well established in the literature [11, 12, 23]. Six monthly dermal injections of PRP in acne scars showed significant changes in scar size in all kinds of scars (*p* < 0.0001) and 40% improvement in the appearance of the skin in literature [7, 8].

In the current study, PRP alone led to a significant reduction in scar volume, increased skin density, minimized pore size, and strengthened the skin barrier. The mechanisms underlying PRP's efficacy in treating acne scars involve the release of various growth factors that promote collagen synthesis, tissue remodeling, and wound healing. These include Platelet‐Derived Growth Factor (PDGF), which stimulates collagen production and tissue repair; Transforming Growth Factor‐Beta (TGF‐β), which promotes collagen deposition and scar remodeling; and Vascular Endothelial Growth Factor (VEGF), which enhances angiogenesis and tissue regeneration [11]. These growth factors work synergistically to improve scar texture and depth.

Fibroblast transplantation is a relatively novel approach for treating dermal depressions such as acne scars, with clinical use dating back nearly two decades [24, 25]. Autologous fibroblasts have demonstrated the ability to promote skin regeneration, rejuvenation, and collagen production, making them valuable for both reparative and esthetic dermatology [26]. Multiple studies have demonstrated the efficacy of fibroblast injections in improving acne scars. A Phase III clinical trial involving 215 patients showed that autologous fibroblast injections produced statistically significant improvements in acne scars compared to placebo, with response rates of 75.0% and 81.6% at 9 and 12 months, respectively [19]. Munavalli et al. conducted a randomized, double‐blind trial involving 99 patients with acne scars and found that three biweekly intradermal injections of autologous fibroblasts significantly improved depressed acne scars compared to a control, with no lasting adverse effects [27]. Fibroblasts synthesize and maintain the extracellular matrix, and secrete growth factors, leading to improved skin texture and reduced scar depth. This process not only fills the depressed areas but also enhances the overall quality of the dermis, resulting in a more even skin surface [25, 26].

Combination therapies are gaining prominence in acne scar management. The combination of PRP with other procedures, such as microneedling or fractional lasers, has been shown to result in higher response rates and enhanced scar improvement [23]. In the current study, adding fibroblasts to PRP led to significantly improved outcomes, including thicker, denser skin, greater scar volume reduction, and a more noticeable decrease in fine pores compared to PRP alone. The combination therapy also resulted in a stronger skin barrier and significant reductions in melanin and erythema indices. Improvements in cutometric skin elasticity parameters (R2, R5, and R7) further suggest potential applications in broader skin rejuvenation.

The autologous nature of fibroblast transplantation eliminates the risk of hypersensitivity reactions that can be associated with earlier treatments such as collagen and hyaluronic acid [15, 16]. Additionally, the continuous collagen synthesis by fibroblast injections has been shown to outperform traditional dermal fillers in terms of duration of effect [19]. A long‐term follow‐up survey, ranging from 36 to 48 months, showed that 70% of patients reported satisfaction with their long‐term results. Furthermore, 88% of these satisfied patients noted continuing improvement of their correction for periods up to 24 months [25]. In a 2008 study, Zhao et al. demonstrated that transplanted fibroblasts survived for up to 5 months in animal models, with significantly increased production of collagen Type III, as confirmed by Sirius Red staining [28]. The addition of PRP may further enhance fibroblast function, potentially boosting their regenerative capacity [29].

To the best of our knowledge, this is the first study to investigate the combined use of fibroblast–PRP therapy for the treatment of atrophic acne scars. No serious adverse effects were observed during the study. Although patient satisfaction was not quantitatively assessed, most participants reported subjective satisfaction with the treatment outcomes.

## 5 Limitations

Although the study demonstrated statistically significant improvements in key parameters, the small sample size (*n* = 8) limits the generalizability of the findings. The observed effects were large enough to achieve significance despite limited power; however, these results should be considered preliminary and require confirmation in larger, adequately powered studies. The short follow‐up period (3 months) is another constraint, as collagen remodeling may continue for up to a year, which could influence the long‐term results. These limitations emphasize the need for future research with a larger sample, longer follow‐up, and objective outcome measures.

## 6 Conclusion

This pilot study provides preliminary evidence that three bi‐weekly sessions of autologous fibroblast injections combined with platelet‐rich plasma (PRP) may offer improved outcomes compared to PRP monotherapy in treating atrophic acne scars. Further larger scale, fully blinded randomized controlled trials with longer follow‐up periods are necessary to validate these results and more clearly define the therapeutic potential of fibroblast‐PRP combination therapy.

## Author Contributions

Contributions to the current study include Sona Zare, Roya Zeinali, Maryam Nouri, Abbas Dehghani, Solmaz Zare, Sharin Khatib, and Mohammad Ali Nilforoushzadeh in the study idea and design, literature review, and drafting and revising the manuscript critically for important intellectual content. Sona Zare, Maryam Nouri, and Mohammad Ali Nilforoushzadeh were involved in conducting the trial, data gathering, drafting the proposal, and obtaining ethical committee approval. Solmaz Zare and Sharin Khatib participated in the literature review, analysis, and drafting the manuscript. Abbas Dehghani made critical revisions to the manuscript. Roya Zeinali conducted literature reviews, drafted the manuscript, and revised the manuscript critically for important intellectual content. Mohammad Ali Nilforoushzadeh serves as corresponding author. All authors have read and approved the final version to be published and agreed to be accountable for all aspects of the work.

## Ethics Statement

All information collected was kept confidential and evaluated without a specific name. Participants in this project adhered to all Helsinki ethical principles. This research was approved by the Research Ethics Committee of Tehran University of Medical Sciences under the ethics code number 1502/130/ص/93 on 2014‐09‐29.

## Consent

All patients provided written informed consent for participation in the study. Patients signed informed consent regarding publishing their data and photographs.

## Conflicts of Interest

The authors declare no conflicts of interest.

## Supporting information


**Table S1:** Biometric indices before and after the intervention of the control group. Due to the nonparametric nature of the test, quartiles are reported alongside the mean and standard deviation.
**Table S2:** Biometric indices before and after the intervention of the case group. Due to the nonparametric nature of the test, quartiles are reported alongside the mean and standard deviation.

## Data Availability

All data supporting the results of this study are included in the manuscript, and no additional external data are available. The raw datasets analyzed during the current study are available from the corresponding author on reasonable request.
