# Supplementary material for: Comparison Combination of Autologous Fibroblast Cells Plus Platelet Rich Plasma (PRP) With PRP Alone in Treatment of Atrophic Acne Scars, a Split‐Face Pilot Study With Biometric Assessment
Source: J Cosmet Dermatol. 2025 Aug 25;24(9):e70413. doi: 10.1111/jocd.70413 (PMC12376645; doi:10.1111/jocd.70413)
Supplement: Supplementary file 1 — Table S1: Biometric indices before and after the intervention of the control group. Due to the nonparametric nature of the test, quartiles are reported alongside the mean and standard deviation. Table S2: Biometric indices before and after the intervention of the case group. Due to the nonparametric nature of the test, quartiles are reported alongside the mean and standard deviation. [file JOCD-24-e70413-s001.docx]

**Supplemental table 1:** Biometric indices before and after the intervention of the Control group. Due to the non-parametric nature of the test, quartiles are reported alongside the mean and standard deviation.

| Parameters |  |  | Percentiles | | |  |
| --- | --- | --- | --- | --- | --- | --- |
|  | **Time** | **Mean±SD** | **25** | **50 (Median)** | **75** | **P-value** |
| TWL | Before | 25.16±7.91 | 19.40 | 24.10 | 33.00 | **0.028*** |
|  | After | 19.11±4.52 | 16.10 | 17.50 | 24.00 |  |
| Corneometer | Before | 47.79±5.7 | 42.07 | 49.20 | 49.70 | 0.866 |
|  | After | 43.43±11.87 | 29.10 | 46.12 | 53.00 |  |
| Melanin | Before | 201.66±66.5 | 144.33 | 194.21 | 254.67 | 0.866 |
|  | After | 204.62±67.27 | 167.00 | 189.14 | 290.00 |  |
| Erythema | Before | 388.3±79.71 | 320.45 | 386.74 | 435.14 | 0.866 |
|  | After | 395.96±79.15 | 312.65 | 389.00 | 473.00 |  |
| Color | Before | 25.43±17.92 | 6.00 | 35.00 | 39.00 | **0.042*** |
|  | After | 29.71±15.6 | 9.00 | 35.00 | 42.00 |  |
| R2 | Before | 0.64±0.08 | 0.59 | 0.64 | 0.68 | 0.575 |
|  | After | 0.66±0.03 | 0.64 | 0.67 | 0.68 |  |
| R5 | Before | 0.50±0.12 | 0.42 | 0.50 | 0.57 | **0.017*** |
|  | After | 0.61±0.12 | 0.50 | 0.64 | 0.70 |  |
| R7 | Before | 0.35±0.08 | 0.29 | 0.34 | 0.38 | 0.161 |
|  | After | 0.37±0.05 | 0.32 | 0.38 | 0.40 |  |
| Skin Thickness | Before | 847.43±195.97 | 640.00 | 901.00 | 1045.00 | **0.043*** |
|  | After | 948.14±182.35 | 890.00 | 988.00 | 1091.00 |  |
| Epidermal Thickness | Before | 74.14±12.32 | 65.00 | 70.00 | 82.00 | 0.310 |
|  | After | 74.29±9.03 | 65.00 | 75.00 | 83.00 |  |
| Dermal Thickness | Before | 773.29±188.44 | 575.00 | 819.00 | 950.00 | 0.063 |
|  | After | 873.86±179.94 | 806.00 | 905.00 | 1023.00 |  |
| Skin Density | Before | 9.76±5.13 | 7.20 | 10.67 | 13.58 | **0.043*** |
|  | After | 10.85±5.26 | 10.25 | 11.10 | 14.54 |  |
| Epidermal Density | Before | 33.37±15.68 | 26.98 | 38.50 | 44.47 | **0.018*** |
|  | After | 37.34±16.62 | 39.41 | 41.33 | 47.54 |  |
| Dermal Density | Before | 7.63±4.02 | 5.64 | 8.12 | 10.72 | **0.028*** |
|  | After | 8.69±4.33 | 7.68 | 8.31 | 12.65 |  |
| Fine Pore | Before | 122.60±57.06 | 77.00 | 132.00 | 163.50 | **0.043*** |
|  | After | 101.40±55.15 | 59.00 | 99.00 | 145.00 |  |
| Large Pore | Before | 33.40±19.71 | 15.50 | 35.00 | 50.50 | **0.043*** |
|  | After | 17.00±11.11 | 5.50 | 22.00 | 26.00 |  |
| Spot | Before | 1.80±1.10 | 1.00 | 2.00 | 2.50 | **0.063*** |
|  | After | 0.60±0.55 | 0.00 | 1.00 | 1.00 |  |
| Scar Acne Volume | Before | 209.25±72.18 | 145.58 | 186.64 | 284.23 | **0.043*** |
|  | After | 153.50±57.31 | 101.90 | 144.40 | 209.64 |  |
| Scar Acne Area | Before | 20.85±6.70 | 15.37 | 18.05 | 27.72 | **0.043*** |
|  | After | 15.44±5.40 | 10.70 | 14.42 | 20.70 |  |
| Scar Acne Depth | Before | 9.60±0.89 | 9.00 | 10.00 | 10.00 | 0.705 |
|  | After | 9.40±0.55 | 9.00 | 9.00 | 10.00 |  |

* A p-value of less than 0.05 was considered statistically significant. TEWL: trans-epidermal water loss; R2: the ability to return to the original position; R5 the elastic part of the suction phase; and R7 the portion of the elastic recovery compared to the complete curve.

**Supplemental table 2:** Biometric indices before and after the intervention of the Case group. Due to the non-parametric nature of the test, quartiles are reported alongside the mean and standard deviation.

| Parameters | Time | Mean±SD | Percentiles | | | P-value |
| --- | --- | --- | --- | --- | --- | --- |
|  |  |  | **25** | **50 (Median)** | **75** |  |
| TWL | Before | 25.51±8.03 | 19.80 | 24.40 | 33.40 | **0.028*** |
|  | After | 17.69±3.97 | 14.20 | 16.80 | 21.30 |  |
| Corneometer | Before | 47.13±5.62 | 42.07 | 48.43 | 48.53 | 1.000 |
|  | After | 44.79±11.97 | 30.23 | 48.33 | 54.23 |  |
| Melanin | Before | 203.24±65.85 | 145.33 | 197.33 | 254.67 | 1.000 |
|  | After | 202.38±67.4 | 161.33 | 186.67 | 288.00 |  |
| Erythema | Before | 390.81±78.59 | 327.33 | 386.00 | 440.33 | 1.000 |
|  | After | 390.05±78.78 | 311.67 | 384.67 | 469.67 |  |
| Color | Before | 25.43±17.92 | 6.00 | 35.00 | 39.00 | **0.027*** |
|  | After | 32.57±15.04 | 12.00 | 35.00 | 45.00 |  |
| R2 | Before | 0.64±0.08 | 0.59 | 0.64 | 0.68 | 0.401 |
|  | After | 0.67±0.04 | 0.65 | 0.68 | 0.71 |  |
| R5 | Before | 0.5±0.12 | 0.41 | 0.50 | 0.57 | **0.017*** |
|  | After | 0.63±0.12 | 0.53 | 0.65 | 0.72 |  |
| R7 | Before | 0.34±0.08 | 0.28 | 0.34 | 0.37 | 0.069 |
|  | After | 0.38±0.04 | 0.35 | 0.39 | 0.41 |  |
| Skin Thickness | Before | 824.57±210.97 | 584.00 | 862.00 | 1041.00 | **0.018*** |
|  | After | 1050.43±96.10 | 966.00 | 1084.00 | 1097.00 |  |
| Epidermal Thickness | Before | 72.86±12.67 | 63.00 | 66.00 | 81.00 | 0.249 |
|  | After | 83.00±16.92 | 72.00 | 81.00 | 85.00 |  |
| Dermal Thickness | Before | 751.71±202.80 | 525.00 | 781.00 | 947.00 | **0.028*** |
|  | After | 967.43±104.83 | 894.00 | 1012.00 | 1019.00 |  |
| Skin Density | Before | 9.49±5.02 | 6.76 | 10.24 | 13.02 | **0.018*** |
|  | After | 12.25±5.77 | 11.77 | 13.25 | 15.56 |  |
| Epidermal Density | Before | 32.45±15.27 | 26.61 | 38.50 | 43.53 | **0.018*** |
|  | After | 41.10±18.29 | 43.40 | 45.33 | 51.21 |  |
| Dermal Density | Before | 7.21±4.24 | 4.54 | 8.05 | 10.81 | **0.018*** |
|  | After | 9.96±4.83 | 8.84 | 10.31 | 13.63 |  |
| Fine Pore | Before | 184.80±124.30 | 80.00 | 190.00 | 287.00 | **0.043*** |
|  | After | 91.00±53.22 | 54.50 | 82.00 | 132.00 |  |
| Large Pore | Before | 45.80±34.90 | 17.50 | 30.00 | 82.00 | **0.043*** |
|  | After | 18.40±11.55 | 8.00 | 15.00 | 30.50 |  |
| Spot | Before | 4.00±3.81 | 1.00 | 3.00 | 7.50 | 0.066 |
|  | After | 1.80±2.95 | 0.00 | 1.00 | 4.00 |  |
| Scar Acne Volume | Before | 233.92±96.67 | 164.83 | 217.53 | 311.21 | **0.043*** |
|  | After | 147.23±82.48 | 79.47 | 132.86 | 222.17 |  |
| Scar Acne Area | Before | 22.54±7.19 | 16.70 | 22.67 | 28.31 | **0.043*** |
|  | After | 13.88±6.68 | 8.04 | 12.96 | 20.18 |  |
| Scar Acne Depth | Before | 9.40±0.89 | 9.00 | 9.00 | 10.00 | 0.317 |
|  | After | 9.60±0.89 | 9.00 | 9.00 | 10.50 |  |

* A p-value of less than 0.05 was considered statistically significant. TEWL: trans-epidermal water loss; R2: the ability to return to the original position; R5 the elastic part of the suction phase; and R7 the portion of the elastic recovery compared to the complete curve.
